# XAB2 TagSNP Is Associated with the Risk of Gastric Cancer in Chinese Population: A Case–Control Study

**DOI:** 10.3390/ijerph18041494

**Published:** 2021-02-04

**Authors:** Yuning Xie, Yuan Yu, Hongjiao Wu, Hui Gao, Zhenbang Yang, Yi Zhang, Xuemei Zhang

**Affiliations:** 1 School of Public Health, North China University of Science and Technology, Tangshan 063210, China; xyn_0634@foxmail.com (Y.X.); wuhongjiao@stu.ncst.edu.cn (H.W.); huig1992@163.com (H.G.); 2 College of Life Science, North China University of Science and Technology, Tangshan 063210, China; yuyuan5188@163.com; 3 School of Basic Medical Sciences, North China University of Science and Technology, Tangshan 063210, China; bzy108@126.com (Z.Y.); zhangxiaoyi0807@163.com (Y.Z.)

**Keywords:** XAB2, gastric cancer, polymorphism, DNA repair, transcriptional coupled repair

## Abstract

XAB2 protein (xeroderma pigmentosum group A-binding protein 2) plays a significant role in the nucleotide excision repair pathway. Polymorphisms in the XAB2 gene may have an effect on the capability of DNA repair and further contribute to the risk of developing various cancers. In order to investigate the relationship between XAB2 genetic variants and the risk of gastric cancer, we performed a hospital-based case–control study. XAB2 tagSNPs were selected and then genotyped by iPlex Gold Genotyping Assay and Sequenom MassArray. By performing logistic regression analysis, odds ratio (OR) and 95% confidence interval (CI) were used to estimate the association of XAB2 tagSNPs with the risk of gastric cancer. Our results showed that XAB2 rs794078AA genotype was associated with a significantly lower risk of gastric cancer compared with GG genotype with OR (95% CI) of 0.33 (0.12–0.91). Stratified analysis indicated a significantly decreased risk for gastric cancer among smokers with rs794078AA genotype compared with nonsmokers with GG genotype (OR = 0.11, 95% CI = 0.01–0.91, *p* = 0.040). The gene–gene interactions by multifactor dimensionality reduction (MDR) showed that tagSNP rs794078 was the best predictive element for gastric cancers (Testing Bal. Acc = 51.68%, *p* = 0.055, cross-validation consistency = 9). Therefore, the XAB2 tagSNP rs794078 may play an important role in the development of gastric cancer.

## 1. Introduction

Gastric cancer is the fourth most common cancer and the third cause of cancer-related death in China [1]. Although the incidence of gastric cancer has reduced rapidly year by year, it still remains one of major health problems [2]. Many environmental factors contribute to the occurrence of gastric cancer, such as dietary habits, tobacco smoking, and bacterial infection [3]. Besides these acquired factors, the genetic difference of individuals also contributes to the risk of gastric cancer [4,5].

The DNA repair system plays a pivotal role in the maintenance of genomic stability by rapidly giving rapid response to DNA damage. Studies have demonstrated that the decline or deficiency of DNA repair capacity could lead to DNA mutation and further cause various cancers, such as squamous cell carcinoma of head and neck, breast carcinoma and gastric cancer [6,7,8]. The human DNA repair system which consists of over 130 genes, can be divided into four major repair pathways: nucleotide excision repair (NER), mismatch repair (MMR), base excision repair (BER), and double-strand break repair (DSBR) [9,10]. By removing various DNA lesions through “cut-patch” mechanism, NER helps DNA resisting the negative impact of mutation [11,12]. There are two subpathways of NER, transcription-coupled NER (TCR-NER) and global genome NER (GG-NER). TC-NER rapidly removes lesions during transcription and GG-NER repairs the damage throughout the genome [8]. The functional polymorphisms in key components of NER could affect DNA repair capacity and further contribute to the development of various cancers. For example, ERCC1 rs11615 and rs17655 polymorphisms were associated with an increased risk of laryngeal cancer [13] and XPD Lys751Gln variant was associated with an increased risk of digestive tract cancer [14].

XAB2 is involved in the process of both GC-NER and TC-NER by interacting with XPA [15]. In TC-NER, XAB2 interacts with Cockayne syndrome groups A (CSA), Cockayne syndrome groups B (CSB), as well as RNA polymerase II to initial the DNA repair [16]. XAB2 is also involved in pre-mRNA splicing, which leads to preimplantation lethality in vitro and in vivo studies [17]. The downregulation of XAB2 could result in RNA synthesis interference and the decrease of mRNA splicing efficiency [18]. Due to the important roles of XAB2 in NER, the functional genetic variants in XAB2 may lower DNA repair capability and further contribute to the occurrence of cancer. In order to investigate the role of XAB2 genetic variations in the development of gastric cancer, tagSNPs of XAB2 were selected and surveyed in this hospital-based case–control study.

## 2. Materials and Methods

### 2.1. Study Population

In this hospital-based case–control study, 500 patients with gastric cancer and 500 cancer-free controls were included. Both patients and healthy controls are all Han Chinese residents. Gastric cancer individuals were recruited at Affiliated Tangshan Gongren Hospital of North China University of Science and Technology (Tangshan, China) between January 2008 and December 2013 before treated with any radiotherapy or chemotherapy. There was no restriction in regard to age, sex, tumor stage and histology type. The controls were randomly selected from a pool of cancer-free population by cancer-screening program conducted in the same region. The healthy controls were frequency matched with cases by age (±5 years) and sex.

The included eligible cancer-free controls had no history of cancer and digestive disease, while cases were newly diagnosed as gastric cancer and had no other malignancies. This study was approved by the institutional review board from the Human Ethics Review Committee of North China University of Science and Technology (12-002). The detailed information about volunteers’ gender, age, and smoking status were also collected after obtaining the informed consent of each subject. In this study, smoker status was defined as participant having smoked more than 100 cigarettes during their lifetime.

### 2.2. TagSNPs Selection and Genotype

To select tag SNPs of XAB2, the HapMap database (HapMap Data Rel27/PhaseII+III, on NCBI B36 assembly, dbSNP b126) was discovered. The selection criteria included that γ^2^ ≥ 0.8 for all SNPs with a minor allele frequency (MAF) ≥ 0.05 based on pairwise linkage disequilibrium (LD) information.

Each participant donated 2 mL of peripheral blood lymphocytes for DNA extraction. Peripheral blood lymphocytes were digested by proteinase K and then used for DNA extraction by genomic DNA extraction kit (DP348) (Tiangen, Beijing, China) according to the instruction. Ultramicro-spectrophotometer MD2000D (Biofuture, UK) was used to detect DNA concentration and purity.

All genotyping was performed with iPlex Gold Genotyping Assay and Sequenom MassArray (Sequenom, San Diego, CA, USA) at BomiaoTech (Beijing, China). Polymerase chain reactions (PCR) and extension primers for each SNP were designed by Sequenom’s MassArray Designer. Briefly, PCR reaction was running in 1× reaction buffer with 5 mM Mg^2+^, 0.5 mM dNTPs, 0.2 U Hotstar DNA polymerase, 0.2 μM forward primer/reverse primer, and 10–50 ng DNA sample. The thermal cycle condition was denaturation at 94 °C for 15 min, followed by 45 cycles of 94 °C for 20 s, 56 °C for 30 s, 72 °C for 1 min, and a final extension at 72 °C for 3 min. PCR products were digested by Shrimp alkaline phosphate (SAP) and then analyzed by SpectroCHIP bioarray (Sequenom, San Diego, CA, USA) and Matrix-assisted laser desorption ionization-time of flight mass spectrometry (MALDI-TOF MS) (Sequenom, San Diego, CA, USA).

### 2.3. Statistical Analysis

The differences of demographic variables and the distribution of genotypes between gastric cancer patients and controls were estimated by two-sided chi-square test. Hardy–Weinberg equilibrium of each SNP in controls was tested by goodness-of-fit chi-square test. By performing multivariate logistic regression analysis adjusted by age, gender and smoking status, odds ratio (OR) and 95% confidence interval (CI) were calculated to assess the association of XAB2 genetic variants with the risk of gastric cancer. The nonparametric multifactor dimensionality reduction (MDR) was employed to determine gene–gene interaction [19]. Testing accuracy higher than 50% indicated a meaningful result. The cross-validation consistency (CVC) presents the number of times a particular combination of factors identified in the same best model. All tests were performed with SPSS software package (version 23.0; IBM, Armonk, NY, USA), and *p* < 0.05 was used as the criterion of significant differences in all statistical analysis.

## 3. Results

### 3.1. Baseline Characteristics of Study Population

The basic information of 500 cases and 500 controls are summarized in Table 1. There were 74.4% males and 28.6% females among cases, and 68.8% males and 31.2% females among controls. No statistically significant differences were found in the distributions of gender between cases and controls (*p* = 0.407). There were 58.8% and 60.4% of nonsmokers and 41.2% and 39.6% of smokers in cases and controls (*p* = 0.652), respectively.

### 3.2. Selected SNPs and the Risk of Gastric Cancer

Using HaploView program, five tagSNPs (rs4134860 T > C, rs794078 G > A, rs794083 C > G, rs4134816 T > C, rs4134819 A > G) were selected for further study. All of selected tagSNPs were intron variants except for one synonymous variant (rs794078). The genotype frequency of each SNP in controls was in agreement with the Hardy–Weinberg equilibrium (*p* > 0.05). The observed genotype frequencies in participants and the association of genotypes with gastric cancer are presented in Table 2.

Of all selected SNPs in XAB2, only one polymorphism was identified to be associated with the risk of gastric cancer. For XAB2 rs794078 G > A polymorphism, we found that AA genotype carriers had a significantly decreased risk for developing gastric cancer (OR = 0.33; 95% CI = 0.12–0.91) in comparison to those with GG genotype. The rs794078 heterozygous AG was not associated with the risk of gastric cancer with OR (95% CI) of 1.29 (0.95–1.76). We also evaluated the association of XAB2 rs794078A allele with the susceptibility to gastric cancer when compared with rs794078G allele and there was no significant association was found. For other tagSNPs, we did not find that they were associated with the risk of gastric cancer.

Multifactor dimensionality reduction (MDR) was used to further investigate gene–gene interaction (Table 3). XAB2 rs794078, rs794083, rs4134819 interaction model indicated high testing balance accuracy; however, which was still lower than rs794078, rs4134819 interaction model. The representative interaction model was rs794078, which was the best predictive factor for gastric cancer (Testing Bal. Acc = 51.68%, *p* = 0.055, cross-validation consistency = 9).

### 3.3. XAB2 rs794078 Variant and Gastric Cancer by Smoking Status

To evaluate the effect of environmental factors on the association of XAB2 polymorphisms with the risk of gastric cancer, an unconditional multivariate logistic regression model was performed. Our data showed that the smokers with XAB2 rs794078 AA genotype had a significantly decreased risk of gastric cancer compared with nonsmokers with GG genotype (OR = 0.11, 95% CI = 0.11–0.91, *p* = 0.040), suggesting rs794078 AA genotype was a protective factor for gastric cancer in smokers (Table 4). We did not find that rs794078 AG genotype affected the risk of gastric cancer when stratified by smoking status.

## 4. Discussion

As a key regulator of NER, XPA played an important role in recognition of DNA lesion and recruitment of other NER proteins. XAB2 was defined as XPA-interacting protein, which consists of 15 tetratricopeptide repeats with 855 amino acids. XAB2 has been purified by means of combination with XPA in the yeast two-hybrid system. XAB2 inhibited all-trans retinoic acid (ATRA)-induced cellular differentiation by interacting with the transcriptional corepressor complexes [20]. XAB2 was also involved in DNA repair system in the form of complex, which included aquarius intron-binding spliceosomal factor (hAquarius), pre-mRNA processing factor 19 (hPRP19), zinc finger protein 830 (ZNF830), and peptidylprolyl isomerase E (PPIE) [15]. A large number of studies have confirmed that XAB2 plays a crucial role in the NER pathway, TC-NER and transcription itself [17,21].

Both genetic variation and gene–environment interaction contributed to personalized susceptibility to gastric cancer [3]. Many studies indicated tagSNPs were associated with gastric cancer. For instance, decay-accelerating factor tag SNP (rs10746463) was associated with the risk of gastric cancer [22]. Polymorphisms in key components of NER pathway were also associated with the risk of gastric cancer and other cancers. For instance, ERCC5 rs1047768 variant reduced the risk of gastric cancer [23], ERCC3 rs4150403 was associated with an increased susceptibility to head and neck cancer in Caucasians, and ERCC6 rs4253132 polymorphism decreased the risk of head and neck cancer among African Americans [24]. In this study, we found that XAB2 tagSNP rs794078AA genotype was associated with a decreased risk of gastric cancer in the Chinese population. However, the rs7940789 A allele was not related to the susceptibility to gastric cancer. This result proved the important role of DNA repair gene in the development of gastric cancer.

Cigarette contains carcinogens such as 4-aminobiphenyl and nitric oxide, which could cause DNA damage and further contribute to cancer occurrence [25]. Cigarette smoking, nicotine and nicotine-derived nitrosamine could lead to gastrointestinal cancer [25]. Recent research has shown that smoking was considered to be the major causal factor of gastric cancer [26]. Several studies demonstrated the interaction of SNPs with smoking status in the development of certain cancer, but the effects were not consistent. For instance, smokers with decay-accelerating factor rs10746463 A allele containing genotype have increased risk of gastric cancer [22]. Tumor necrosis factor α (TNF-α)-1031 T/C, -863 C/A and -857 C/T were also associated with higher risk of gastric cancer among smokers [27]. In our study, smokers with rs794078 G > A variant had a significantly decreased risk of gastric cancer. This finding demonstrated a reverse association between XAB2 rs794078 and gastric cancer risk in the Chinese population. This result was in accordance with the study of the CYP1A1 polymorphism, which showed that smokers with T6235C transition polymorphism have a significantly lower risk of developing gastric cancer [28].

To date, there are few studies to demonstrate the association of XAB2 polymorphism with the gastric cancer susceptibility. Our finding provided the first evidence that XAB2 rs794078AA genotype acted as a protective factor of gastric cancer. The XAB2 polymorphism might be a biomarker for risk prediction of gastric cancer in the future. However, our study has its limitations. As XAB2 rs794078AA genotype has low frequency, a large population is still needed to verify our current findings.

## 5. Conclusions

In conclusion, our results demonstrated that XAB2 rs794078 was associated with the risk of gastric cancer, which indicated that natural occurring genetic variation in key genes of NER may underlie the susceptibility to cancer.

## Figures and Tables

**Table 1:** Distribution of selected characteristics in cases and controls.

Variables	Cases (*n* = 500)		Controls (*n* = 500)		*p* Value
	No	(%)	No	(%)	
Sex					0.407
Male	357	71.4	344	68.8	
Female	143	28.6	156	31.2	
Age					0.317
≤50	141	28.2	156	31.2	
51–60	149	29.8	157	31.4	
>60	210	42.0	187	37.4	
Smoking status					0.652
Nonsmoker	294	58.8	302	60.4	
Smoker	206	41.2	198	39.6	

**Table 2:** Genotype frequencies of XAB2 among cases and controls and their association with gastric cancer.

Genotypes	Controls (*n* = 500)		Cases (*n* = 500)		OR (95% CI) ^§^	*p* Value
	No	(%)	No	(%)		
rs4134860						
TT	363	72.6	350	70		
CT	127	25.4	136	27.2	1.10 (0.83–1.46)	0.51
CC	10	2	14	2.8	1.47 (0.64–3.37)	0.367
rs794078						
GG	392	78.4	378	75.6		
AG	93	18.6	117	23.4	1.29 (0.95–1.76)	0.105
AA	15	3	5	1	0.33 (0.12–0.91)	0.032
rs794083						
CC	241	48.2	237	47.4		
CG	199	39.8	209	41.8	1.07 (0.82–1.39)	0.638
GG	60	12	54	10.8	0.93 (0.61–1.40)	0.71
rs4134816						
TT	461	92.2	470	94		
CT	39	7.8	30	6	0.81 (0.49–1.34)	0.411
rs4134819						
AA	129	25.8	112	22.4		
AG	246	49.2	253	50.6	1.18 (0.87–1.62)	0.287
GG	125	25	135	27	1.26 (0.89–1.80)	0.199

^§^ Data were calculated by logistic regression and adjusted for age, gender and smoking status.

**Table 3:** Summary of multifactor dimensionality reduction (MDR) gene–gene interaction results for XAB2 gene.

Models	Training Bal.	Testing Bal.	*p* Value	Cross-Validation
	Acc (%)	Acc. (%)		Consistency
rs794078	52.40	51.68	0.055	9/10
rs794078, rs4134819	53.38	50.22	0.623	8/10
rs794078, rs794083, rs4134819	54.29	50.01	0.623	4/10

**Table 4:** Risk of XAB2 rs794078 genotypes with gastric cancer by smoking status.

Genotype	Smoking Status					
	Nonsmoker (Cases/Controls)	OR (95% CI) ^§^	*p* Value	Smoker (Cases/Control)	OR (95% CI) ^§^	*p* Value
GG	229/243	1.00 (reference)		149/149	1.02 (0.74–1.42)	0.891
AG	61/53	1.21 (0.81–1.83)	0.354	56/40	1.47 (0.92–2.34)	0.107
AA	4/6	0.69 (0.19–2.48)	0.567	1/9	0.11 (0.01–0.91)	0.04

^§^ Data were calculated by logistic regression and adjusted for age and gender.

## Data Availability

The data presented in this study are available on request from the corresponding author. The data are not publicly available due to patient privacy.
